# The Relationship between All-Cause Natural Mortality and Copy Number of Mitochondrial DNA in a 15-Year Follow-Up Study

**DOI:** 10.3390/ijms241310469

**Published:** 2023-06-21

**Authors:** Sofia Malyutina, Vladimir Maximov, Olga Chervova, Pavel Orlov, Anastasiya Ivanova, Ekaterina Mazdorova, Andrew Ryabikov, Galina Simonova, Mikhail Voevoda

**Affiliations:** 1 Research Institute of Internal and Preventive Medicine-Branch of Institute of Cytology and Genetics SB RAS, Novosibirsk 630089, Russia; medik11@mail.ru (V.M.); orlovpavel86@gmail.com (P.O.); ivanova_a_a@mail.ru (A.I.); mazdorova@mail.ru (E.M.); a_ryabikov@hotmail.com (A.R.); gsimonova2019@gmail.com (G.S.); mvoevoda@ya.ru (M.V.); 2 UCL Cancer Institute, University College London, London WC1E 6BT, UK; o.chervova@ucl.ac.uk

**Keywords:** mitochondrial DNA copy number (mtDNA-CN), ageing, mortality, circulatory system diseases, cancer, case–control, population

## Abstract

We explored the relationship between the copy number of mitochondrial DNA (mtDNA-CN) and all-cause natural mortality. We examined a random population sample in 2003/2005 (*n* = 9360, men/women, 45–69, the HAPIEE project) and followed up for 15 years. Using a nested case–control design, we selected non-external deaths among those free from baseline cardiovascular diseases (CVD) and cancer (*n* = 371), and a sex- and age-stratified control (*n* = 785). The odds ratios (ORs) of death were 1.06 (95%CI 1.01–1.11) per one-decile decrease in mtDNA-CN independent of age, sex, metabolic factors, smoking, alcohol intake and education. The age–sex-adjusted ORs of death in the second and first tertiles of mtDNA-CN vs. the top tertile were 2.35 (95% CI 1.70–3.26) and 1.59 (1.16–2.17); an increased risk was confined to the second tertile after controlling for smoking and metabolic factors. The multivariable-adjusted OR of CVD death was 1.92 (95% CI 1.18–3.15) in tertile 2 vs. the top tertile of mtDNA-CN, and for cancer-related death the ORs were 3.66 (95% CI 2.21–6.05) and 2.29 (95% CI 1.43–3.68) in tertiles 2 and 1 vs. the top tertile. In the Siberian population cohort, the mtDNA-CN was an inverse predictor of the 15-year risk of natural mortality, due to the greatest impact of CVD and cancer-related death. The findings merit attention for exploring further the role of mtDNA in human ageing and the diversity of mortality.

## 1. Introduction

Worldwide, there are at least 900 million people over 60 years old and, according to the United Nations estimates, the world’s population is expected to reach 8.6 billion people by 2030, of which more than 1.4 billion will be over the age of 60 [1]. According to WHO data, the rate of mortality among the 60+ population for both sexes varied between 3167 and 4597 per 100,000 persons (for high- and low-income territories, correspondingly) [2]. 

A decomposition analysis [3] based on data from the Global Burden of Disease Study (2017) estimated that population ageing was associated with an increase of 12 million deaths worldwide between 1990 and 2017, representing 27.9% of total global deaths. The two largest contributors from population ageing to disease-specific deaths globally between 1990 and 2017 were ischemic heart disease (3.2 million) and stroke (2.2 million).

Globally, the top 10 causes of death defined by WHO in 2019 include ischemic heart disease, stroke, chronic obstructive pulmonary disease (COPD), lower respiratory infections, neonatal conditions, trachea, bronchus and lung cancers, Alzheimer’s disease and dementia, diarrheal diseases, diabetes mellitus and kidney diseases [4].

In the elderly aged 65+, the top 10 underlying causes of death according to CDC data in 2021 were diseases of the heart, malignant neoplasm, COVID-19, cerebrovascular diseases, chronic lower respiratory diseases, Alzheimer’s disease, diabetes mellitus, accidents, kidney diseases and Parkinson’s disease [5]. 

The process of ageing is characterized by a progressive decline in organism functions, which leads to multimorbidity and mortality. To respond to the increase in deaths related to population ageing for the leading causes of death, ageing-related health research represents the emergent agenda. There is a battery of molecular markers of “biological age” which are explored as determinants of the rate of ageing, including the copy number of mitochondrial DNA (mtDNA-CN). Mitochondria regulate a number of cellular processes, including ATP production by oxidative phosphorylation (OXPHOS), apoptosis, β-oxidation of fatty acids and the biogenesis of iron–sulfur clusters [6,7], and are involved in the production of reactive oxygen species (ROS) [8]. Ageing is accompanied by a decay in mitochondria function, alteration in its morphology, mitochondrial content and OXPHOS capability [6].

The content of mtDNA in cells and tissues is connected to metabolic activities [9], but how mtDNA copy number (mtDNA-CN) is adjusted to and maintained at a certain level is poorly understood. Many studies showed a reduction in mtDNA-CN in older subjects [6,10,11,12], and the estimates of an extent of decline of copies by decade have not been reported [12]. There are facts of the association between low mtDNA-CN and all-cause and cardiovascular (CVD) mortality [11,13,14]; however, the studies of mtDNA content in nonagenarians and centenarians have contradictory results [15,16]. Referring to specific age-related outcomes, the inverse relationship between mtDNA-CN and fatal and non-fatal CVD outcomes was reported in several studies [13,14,17,18]. At the same time, the estimates of potential associations between an alteration in mtDNA-CN and chronic kidney disease [19,20,21] or cancer [22,23,24] are rather heterogeneous depending on the cancer type and study design. 

Using a population cohort established in Novosibirsk in the frame of the HAPIEE study (Health, Alcohol and Psychosocial Factors in Eastern Europe) in 2003–2005 with longitudinal follow-up, we expanded the study to investigate the association between biomarkers of ageing (DNA methylation, leukocyte telomere length, and mitochondrial DNA copy number) and the risk of a number of age-related outcomes. The results obtained so far have been reported elsewhere [25,26,27,28,29]. 

Being a part of this project, the present paper is aimed to explore the relationship between the copy number of mitochondrial DNA and all-cause non-external mortality during a 15-year follow-up in a population-based case–control study.

## 2. Results

### 2.1. General Baseline Characteristics of the Studied Groups

At baseline, we examined a random population sample from 2003/2005 (*n* = 9360, men/women, 45–69, the HAPIEE project) and followed up for 15 years. Using a nested case–control design, we selected non-external deaths among those free from baseline cardiovascular diseases (CVD) and cancer (*n* = 371), and a sex- and age-stratified control (*n* = 785). The general baseline characteristics of the cases and controls are shown in Table 1. 

The subjects in the group of death cases, at baseline, were somewhat older; had a higher blood pressure (BP), heart rate, concentration of serum TG and fasting plasma glucose (FPG) and values of waist-hip ratio (WHR) and body mass index (BMI); and also those who were deceased had more common hypertension and diabetes mellitus type 2, were more commonly smokers and had lower levels of education compared to the control group; women in the death group were more frequently in menopause status. 

In the case group, the causes of death were clustered as follows: the proportion of diseases of the circulatory system [ICD-10: I00–I99] comprised 51.8%; among them, 36.6% were by coronary heart disease [ICD-10: I20–I25], 35.5% were from neoplasms [ICD-10: C18–C20] and 12.7% were other reasons. The distribution of death–cause categories in the selected death cases was close to the distribution in the entire cohort sample of deceased with known death causes (*n* = 2217). 

The mean (SD; median) of the age of death as registered was 68.9 years (8.33; 69.7); the time period between blood draw and registration of death was 9.29 years (4.36; 10.53). The mean age of death and time to event in the case group were close to the same indicators in the entire cohort sample of deceased (69.4, SD = 7.77 and 7.93, SD = 4.33 years). 

**Figure 1:**
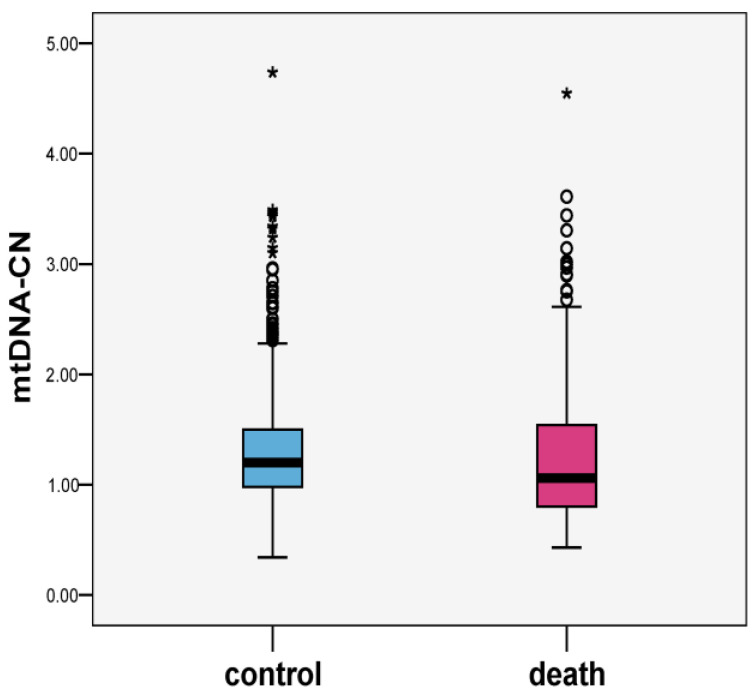
Boxplots of mtDNA-CN values in all-cause death and control groups (*n* = 1156).

**Figure 2:**
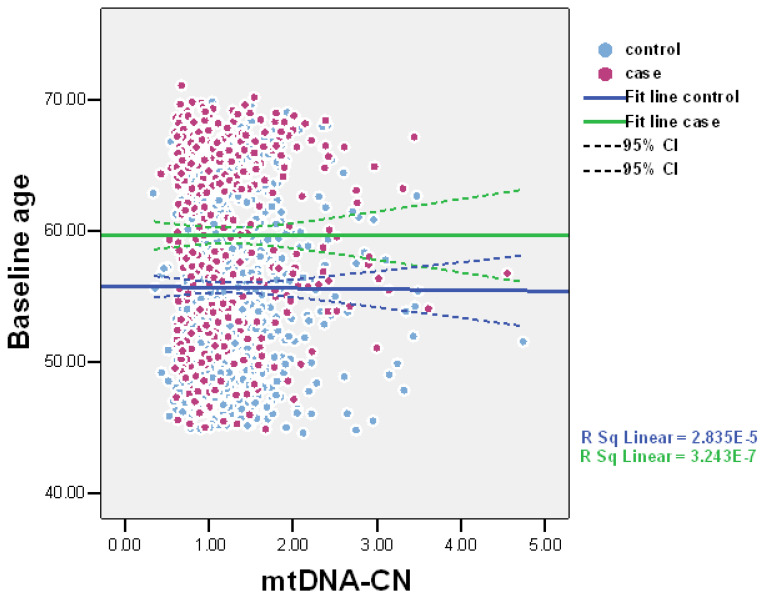
Scatter plots of mtDNA-CN values in all-cause death and control groups (*n* = 1156).

The values of baseline mtDNA-CN were suggestively lower among cases compared to controls: means (SD; median) 1.24 (0.60; 1.06) and 1.30 (0.49; 1.19), *p* = 0.098, respectively (Figure 1). Scatter plots of mtDNA-CN values in the all-cause death and control groups are presented in Figure 2. MtDNA-CN values negatively correlated with baseline age. The correlation coefficient between mtDNA-CN and age was −0.029, *p* = 0.226. 

### 2.2. Association between Baseline mtDNA-CN and Risk of Non-External Death

The measurement of baseline mtDNA-CN was performed with real-time quantitative polymerase chain reaction (qPCR) using StepOnePlus™ (Applied Biosystems, Thermo Fisher Scientific Inc., Waltham, MA, USA) in cases and controls. We estimated the odds ratio of all-cause death per one-decile decrement in mtDNA-CN as a continuous variable in multivariable-adjusted logistic regression. 

Table 2 presents the odds ratios of death per one-decile decrement in baseline mtDNA-CN. In the model adjusted for age and sex, the OR of death per one-decile decrement in mtDNA-CN was 1.08 (95% CI 1.04–1.13). Similarly, in fully adjusted Model 3, the association remained modestly significant, the OR was 1.06 (95% CI 1.01–1.11) regardless of age, sex, smoking, systolic BP (SBP), total cholesterol (TC), TG, FPG or DM2, BMI or WHR, education and alcohol intake (Table 2). 

Table 2 (at the bottom) shows the coefficients of association separately for men and women. In the age-adjusted model, the relationship between mtDNA-CN and death had very close ORs in men and women compared to the pooled results. In the multivariable-adjusted model, the association remained modestly significant in women, the OR of death was 1.08 (95% CI 1.01–1.17) per one-decile decrement in mtDNA-CN and attenuated to not statistically significant in men.

Also, we estimated the odds ratios of death by tertiles of mtDNA-CN values using the top tertile as a reference in logistic regression, applying the same covariates for models. The tertile cut points of mtDNA-CN were 1.002 and 1.370. The ORs of 15-year risk of death by tertiles of baseline mtDNA-CN are shown in Table 3. 

After controlling for age and sex, the risk of death increased in tertiles 2 and 1 vs. tertile 3: OR = 2.35 (95% CI 1.70–3.26) and 1.59 (1.16–2.17), respectively. In the fully adjusted model, the risk of death remained higher in tertile 2 of mtDNA-CN compared to the highest tertile, the OR=2.50 (95% CI 1.75–3.59); the risk coefficient in tertile 1 was also positive but statistically not significant with OR = 1.36 (0.96–1.91), *p* for trends = 0.064 (Table 3). Similarly, both in men and women, the ORs of death in tertile 2 of mtDNA-CN were more than two times higher vs. tertile 3 while the ORs in tertile 1 did not reach a statistically significant level.

### 2.3. Association between Baseline mtDNA-CN and Risk of of Cause-Specific Death 

For sensitivity analyses, we evaluated the relationship between baseline mtDNA-CN and death in three groups by the cause of death separately (Table 4 and Table 5). 

The association was the strongest for the risk of fatal cancer with an OR of 1.20 (95% CI 1.11–1.29), *p* < 0.001 per one-decile decrease in mtDNA-CN in the fully adjusted model. In the assessment by tertiles of mtDNA-CN, the risk of cancer death was higher in tertile 2 and tertile 1 vs. tertile 3, OR = 3.66 (95% CI 2.21–6.05) and 2.29 (95% CI 1.43–3.68). The risk of CVD death increased only in tertile 2 compared with the highest tertile, the OR = 1.92 (95% CI 1.18–3.15) independent of other factors. The relationships between baseline mtDNA-CN and 15-year risk of death from “other” reasons were insignificant both per decile and per tertile categories of mtDNA-CN value (Table 4 and Table 5). 

### 2.4. Other Sensitivity Analyses

In further sensitivity analysis, we estimated the association between death and baseline mtDNA-CN values, excluding early fatal events that occurred during the first two years since entering the cohort. The results were similar but somewhat weaker with an OR of death of 1.06 (95%CI 1.01–1.12) per one-decile decrease in baseline mtDNA-CN (Supplementary Materials, Table S1). Then, we repeated the analysis excluding fatal events that occurred during the first ten years (below the median of the time between the baseline and death) after the baseline. The results attenuated further to borderline level with an OR of death of 1.05 (95%CI 0.99–1.12) per one-decile decrement in baseline mtDNA-CN (Supplementary Materials, Table S1). Finally, in the secondary analysis to ensure robustness, we tested the association between death and mtDNA-CN values in the entire dataset of death without applying any exclusion criteria (all causes of death and not excluding prevalent CVD or cancer) against of those who did not die by censoring the date and using available mtDNA-CN measurements (*n* = 1474 totally). In age- and sex-adjusted models, the results were similar (OR = 1.05; 95% CI 1.01–1.10) to those in the primarily selected nested case–control dataset and were attenuated after controlling for smoking and metabolic factors.

## 3. Discussion

In a population cohort (Novosibirsk, Russia), we conducted a nested case–control study including cases of natural death from all causes except external reasons that occurred during a 15-year follow-up and age and sex frequency-matched controls. Those with lower baseline mtDNA-CN had a modestly increased 15-year risk of all-cause natural death with an adjusted OR of 1.06 per one-decile decrement in mtDNA-CN value and the risk of death was 2.50 in the second tertile of mtDNA-CN vs. the top tertile independent of age, cardiometabolic factors, smoking, alcohol intake, and education. Both in men and women, the ORs of death in tertile 2 of mtDNA-CN were more than two times higher vs. tertile 3, while the ORs in tertile 1 were also positive but statistically not significant. After stratification by the causes of death, the strongest inverse association between death and mtDNA-CN was observed for cancer-related death, with an adjusted OR 1.20 per one-decile decrease in mtDNA-CN and a 2–3.5 times increased risk in two low tertiles against the top tertile of mtDNA-CN. The risk of CVD death increased two times in the middle tertile vs. the top tertile of mtDNA-CN value, independent of other factors. 

Our findings of an inverse relationship between mtDNA-CN and all-cause mortality are consistent with the Danish study of GEMINAKAR, the MADT, the LSADT cohorts of siblings and the 1905 birth cohort [11]; ARIC and CHS cohorts [13]; a case–control CAVASIC Study [14]; a prospective WHILA study of women in Sweden [30]; and are in line with the genome-wide analysis (GWA) of mtDNA-CN in the CHARGE Consortium and UK Biobank [31]. A long-term Danish study of twins and siblings (*n* = 1067, 891 analyzed for mortality; follow-up from 1995/1998 to 31 December 2012) pointed at the inverse association of mtDNA-CN with age and all-cause mortality, as well as with a decline in cognitive and physical functions. The stratified data showed a 17% lower risk of dying in those in the 2nd–4th quartiles of mtDNA-CN versus the 1st quartile (OR = 0.83; 95% CI 0.71–0.98) [11]. Ashar F.N. et al., 2015, explored two large multi-ethnic cohorts (ARIC and CHS; *n* = 16,401; >7000 deaths) in a prospective design. The authors found a strong association between mtDNA copy number and age, sex, frailty and mortality risk with an Hazard Ratio (HR) = 1.47 (95% CI 1.33–1.62) for the lowest quintile versus the highest quintile (pooled cohorts) [13]. In a smaller case–control study of subjects with peripheral arterial disease (PAD) against control (CAVASIC; 236 cases/249 controls; 37 deaths; 7-year follow-up), the patients in the lowest quartile had an adjusted HR of 2.66 (95% CI 1.27–5.58) for all-cause mortality compared with combined 2–4 quartiles [14]. In a prospective WHILA study (*n* = 2508, women, 17 years follow-up), baseline mtDNA-CN was inversely related to overall mortality with an HR 1.27 (95% CI 1.10–1.40) for the lowest quartile vs. the top quartile [30]. The GWA of mtDNA-CN based on CHARGE Consortium and UK Biobank cohorts (*n* = 24,622; median follow-up 4318 days) found a nominally significant association between mtDNA haplotypes and the overall non-external mortality (*p* = 0.044) [31].

At the same time, the relatively small InCHIANTY study (*n* = 627; population cohort; 6-year follow-up) showed the diverse associations between mtDNA-CN and death for nondiabetics and diabetics and reported the positive relationship of mtDNA-CN with mortality (β = 1.52) in models including the interaction terms (DM2 and biomarker) [32]. The directions of associations are consistent with the view that increased mtDNA-CN in diabetics may reflect an aggregation of DNA from dysfunctional organelles.

Further, we considered the relationship between mtDNA content and cause-specific death. Our results of the inverse association between mtDNA-CN and CVD death are in line with the findings from the above-mentioned ARIC and CHS cohorts [13] and the case–control study of PAD patients, CAVASIC [14]. These data are further supported by a consistently observed inverse association between mtDNA-CN and prevalent or incident CVD [17,30,33], also summarized in meta-analysis by Yue P, 2018 [34]. The negative relationship specifically for sudden cardiac death (SCD) was shown by Znang et al. in the ARIC study (*n* = 11,093; 361 SCD; 20.4 years of follow-up) with an HR for SCD of 2.24 (95%CI 1.58–3.19) in the 1st vs. 5th quintiles of mtDNA-CN [18]. Interestingly, in our dataset, the risk of CVD death was confined to the middle tertile of the mtDNA-CN value. This finding differs from the approximately linear association with SCD over the range of mtDNA-CN values observed in the ARIC study [18]; the absence of a dose–response effect may be partly related to the heterogeneity of CVD deaths due to various circulatory diseases and the moderate total sample in our observation. 

Our results of the inverse association between mtDNA-CN and cancer related death are in line with a recent prospective Swedish study [24], partly in line with the Shanghai Women’s Health Study of gastric and colorectal cancer [35,36], and with a small case-control study of lung cancer in China [37]. In a prospective Swedish study (*n* = 3325; women; 15.2 years follow-up), baseline mtDNA-CN was inversely associated with all-cause (HR = 1.2) and pooled cancer-related mortality (HR = 1.21) per 1 SD decrease in mtDNA-CN (mainly due to impact of colorectal and genital cancer) [24]. In the Shanghai Women’s Health Study in a case–control design, inverse associations were shown between baseline mtDNA-CN and risk of gastric [36] and colorectal cancer [35]. In a case–control study in China, lower mtDNA-CN was associated with poorer lung cancer prognosis [37]. On the other hand, in a meta-analysis (38 studies; 668/9923 cancer cases/controls), Mi et al., 2015, did not observe a significant association between mtDNA-CN and pooled cancers, with the exception of the direct association with lymphoma and inverse association with skeleton cancer [38]. Another meta-analysis (18 studies, largely Asian, *n* = 3961) estimated that for dichotomized categories, a high mtDNA-CN level in blood vs. low predicted a poor prognosis for overall survival (HR = 1.624, 95% CI: 1.211–2.177), while high mtDNA-CN in tumor tissue predicted a better outcome (HR = 0.604 95% CI: 0.406–0.899) [39]. GWA of mtDNA-CN (CHARGE Consortium; UK Biobank) did not reveal an association between mtDNA haplotypes and cancer mortality [31]. Van Osch et al., 2015, found decreased mtDNA-CN in colorectal cancer tissue compared to resected, and an inverse U-shaped relationship between colorectal cancer survival and mtDNA-CN [40].

Due to the integral position of mitochondria in cellular metabolism, mitochondrial dysfunction plays a critical role in the pathways of several ageing-related diseases. Correlative studies of degenerative diseases have strongly implicated mtDNA mutations and OXPHOS dysfunction in these conditions; mtDNA-CN is considered as a proxy for mitochondria function [41], but the precise mechanisms of association between mtDNA-CN and overall mortality/underlying diseases are largely unclear. 

Regarding CVD, several pathways were shown connecting decreased mtDNA-CN and arrhythmogenesis: compromised ATP production and energy supply to ion channels leads to membrane instability [18,42,43]; excessive ROS production can alter action potential and cardiac excitability [42] and trigger the opening of mitochondrial channels with further “mitochondrial ROS-induced ROS release” [44]; and regional mitochondria depolarization with the activation of K^+^ currents forms a metabolic sink contributing to the re-entry. It was shown that mtDNA that escapes autophagy leads to an inflammatory response and may impact myocarditis, cardiomyopathy and heart failure [30,45]. There is evidence of mitochondrial dysfunction in atherosclerosis: in human plaques, decreased mtDNA and impaired respiration were shown; inflammation and excessive ROS production are known proatherogenic factors promoting lipid oxidation, the uptake of inflammatory cells to arterial wall, SMC proliferation and cytokine release [14,45]; and disturbances of the electron transport chain with reduced ATP content can promote apoptosis, which, together with inflammation, impacts plaque rupture [14]. 

The estimates of associations between alteration in mtDNA-CN and cancer are rather heterogonous. MtDNA-CN differs between cancer and non-affected tissue in several cancer types [24], and the impact of mitochondria metabolism on tumor onset and progression is heterogeneous by cancer type [6]. In The Cancer Genome Atlas project, Resnick et al., 2015, showed that seven cancer types had decreased mtDNA copies in tumor cells (bladder, breast, esophageal, head/neck squamous cell, kidney and liver), one had increased mtDNA copies (lung adenocarcinoma) and seven had no difference from normal mtDNA content (colorectal, kidney, pancreatic, prostate, stomach, thyroid and uterine). Referring to survival, mtDNA content in different tumors was associated with better or poor survival [23]. Existing data suggest that the mtDNA-CN changes depend on mutations in nuclear or mtDNA and serve as an adaptive response toward these mutations for certain cancer types [6,46]. A recent report supports the role of mtDNA mutations in OXPHOS defects facilitating intestinal tumor [47]. Somatic mutations in the mtDNA control region (D-loop) are among the most studied mtDNA cancer variants [22]; they may be shaped by tumor-specific pressure and involved in tumorogenesis [48] and have been linked to cancer prognosis. Mutations in this region mediate mtDNA replication and transcription and can affect mtDNA copy number [22]. During the ageing process, telomere attrition is involved in the regulation of mitochondrial genesis and function; specifically, the role of the telomere-p53-mitochondrial axis for cancer has been shown [24]. Additionally, recent data from ARIC, CHS and Framingham cohorts provide evidence that the changes in mtDNA-CN influence nuclear DNA methylation, resulting in differential gene expression that may contribute to disease and mortality via altered cell signaling [49].

### Study Limitations and Strengths

The reported study results should be considered taking into account their potential limitations. The sample size is moderate (*n* = 1156). However, this case–control analysis included the random age- and sex-stratified set of natural deaths (excluding death from external causes) that occurred in a large cohort (9360) within a long-term follow-up of 15 years. We ensured the completeness of mortality registration data by collecting information from the overlapping sources (Mortality Register; CVD, Cancer, and Diabetes Registers; proxy information about deceased participants obtained via relatives/address bureau during two postal interviews and two repeated cohort examinations). In a random sample of death cases selected for this analysis, very few cases were ruled out due to technical reasons (no available DNA material or inadequate quality of mtDNA-CN genotyping). The cause of death was established among 83% of the entire group of diseased participants. The controls satisfied strict exclusion criteria and were frequency-matched to cases by age and sex. Taking all the above together, we consider that the study design represents the pattern of natural death occurrence for the studied population. 

An additional potential limitation might be related to the effect of early death, where the retrospective design or early follow-up may reflect reverse causation. To avoid this shortfall, we used a prospective design among those who were free from prevalent major CVD or cancer at baseline. Furthermore, we carried out two sensitivity analyses excluding death cases that occurred within the first 2 years or within the first 10 years after the baseline blood drawing, which practically did not change the results.

Another concern is the sex-related variance in mtDNA-CN (known higher values in women vs. men). To protect against relevant limitations, we had a similar sex distribution in the case and control groups and the models were sex-adjusted. Additionally, we repeated the analysis separately for men and women. It allowed us to see that the modest inverse association between death and continuous mtDNA-CN value was confined to women. At the same time, in the tertile analysis, the ORs of death were significant both in men and women and of similar direction and values compared to the combined dataset.

About one half of the all-cause natural mortality in our sample was death due to circulatory diseases, one third-due to cancer and the rest from other causes. Considering the diversity of the relationship between mtDNA-CN and death from different causes, we also analyzed the risk of death by a cause-specific approach. The stratified analysis showed a doubled risk of CVD death in the middle tertile of mtDNA-CN compared with the top tertile, and a strong inverse association between mtDNA-CN (as a continuous value or categorized by tertiles) and cancer-related death. Thus, this sensitivity analysis confirmed the general results for the pooled non-external death data. 

Finally, we checked the robustness of the estimates by conducting a secondary analysis based on the wider dataset of death and controls without exclusion of prevalent CVD or cancer and with available mtDNA-CN measurements (*n* = 1474 totally). The results in age- and sex adjusted models were similar to those in the primarily selected nested case–control dataset.

The presented study also has a number of strengths. To our knowledge, this is the first investigation of the association between mtDNA-CN and the risk of natural death in the Russian (Caucasoid) population cohort, as well as in the Eastern European population. It is noteworthy that we investigated the links between this age-related biomarker and cause-specific death.

These findings represent the first evidence of the direction and values of association between mtDNA-CN and all-cause, circulatory-disease- and cancer-related mortality in the studied population, which were previously under-reported. 

## 4. Materials and Methods

### 4.1. Study Population and Design

We examined a random population sample in two administrative districts of Novosibirsk at baseline in 2003/05 (*n* = 9360, age 45–69) and re-examined in 2006/08 and 2015/18 in the HAPIEE Study (Health, Alcohol and Psychosocial Factors in Eastern Europe, http://www.ucl.ac.uk/easteurope/hapiee-cohort.htm, accessed on 15 March 2023). The generated cohort was followed-up until 31 December 2019 for an average of 15.9 (SD 0.74, median 15.9) years for a number of outcomes (myocardial infarction, stroke, cancer, diabetes mellitus) and cause-specific mortality. 

The Information on all-cause and cause-specific mortality was collected in Novosibirsk at the Institute of Internal and Preventive Medicine (IIPM), using overlapping sources: the Population Registration Bureau (ZAGS), Regional Bureau of Medico-legal expertise and the data received at serial examinations (such as contacts with proxies of deceased participants and the address bureau). During a 15-year follow-up period, 2681 deaths were ascertained in the cohort. The causes of death were classified by ICD-10 codes extracted from death certificates. When death certificate or other medical records were not available (e.g., moved from the region/city), the verbal autopsy was applied based on proxy information about the death. 

The specific cause of death was established among 83% (2217) of the entire group of diseased participants (nearly 1% of them by verbal autopsy); a specific death reason was not defined among 17% of those deceased, including 0.3% with an unknown year of death.

### 4.2. Sample Selection 

Present analysis was conducted in a nested case–control study design. The random age- and sex-stratified sample of deaths was selected with the following inclusion criteria: available ICD-10 codes, any cause of death except external, free from prevalent major cardiovascular diseases (CVD) and cancer at baseline and available DNA material (*n* = 400). The universal control group for this study included those alive by the census date (31 December 2019) with the same exclusion criteria. We randomly selected limited controls that were age- and sex-frequency matched to outcome cases (*n* = 806). After exclusion of technically inadequate DNA samples or inappropriate genotyping of mtDNA-CN, finally, a death group of 371 and control groups of 785 were included for the analysis. 

Death causes by ICD-10 were clustered into three categories: diseases of the circulatory system [ICD-10: I00–I99], neoplasms [ICD-10: C18–C20] and other reasons. The distributions of three death cause categories in the selected death cases were close to the distribution in the entire death sample. The general characteristics of the studied groups are summarized in Table 1. The study was conducted in accordance with the relevant ethical guidelines and regulations. All study participants signed informed consent for participation; the study protocols were approved by the Ethical Committee of the Research Institute of Internal and Preventive Medicine-Branch of Federal State Budgeted Research Institution, “Federal Research Center, Institute of Cytology and Genetics, Siberian Branch of the Russian Academy of Sciences” (IIPM-Branch of IC&G SB RAS), Protocol No. 1 from 14 March 2002 and Protocol No. 12 from 8 December 2020.

### 4.3. Data Collection 

The collection of baseline data was conducted within the HAPIEE project. The protocol included a standardized interview, objective examination and the collection of blood samples. We assessed the history of hypertension, diabetes mellitus, cardiovascular and other chronic diseases, health and behavioral factors, socio-economic circumstances, the measurement of blood pressure (BP), anthropometric indices and physical performance. The details of the protocol are reported elsewhere [50]. 

The lifestyle habits, health and socio-economic circumstances were assessed by structured questionnaire. Smoking status was categorized as current smoker (at least one cigarette a day), former smoker and never smoked. The alcohol consumption was categorized into 5 categories by frequency of intake (not drinking, less than 1 occasion/month, 1–3 occasions/month, 1–4 occasions/week, 5+ occasions/week). 

The level of education was categorized into 4 categories (higher (university degree), secondary, vocational and primary or less than primary education). Marital status was dichotomized as married (or cohabiting) and single (never been married, divorced or widowed) in present analysis. 

The height, waist and hip circumference and weight were measured with accuracy to 1 mm and 100 g, respectively, and used in calculating waist–hip ratio (WHR, units) and body mass index (BMI, kg/m^2^). Blood pressure (BP) was measured three times (Omron M-5) on the right arm in a sitting position after a 5 min rest period with 2 min interval between the measures. In this study, we used the average of three BP measurements. 

Blood samples were drawn at a fasting state (at least 8 h after the last meal). Serum was stored in a deep freezer (minus 80 °C). The concentrations of blood serum total cholesterol (TC), triglycerides (TG), high-density lipoprotein cholesterol (HDLC) and glucose were measured enzymatically within one month from sample collection using KoneLab 300i autoanalyser (Thermo Fisher Scientific Inc., USA) with kits from Thermo Fisher Scientific. The Friedewald formula was used for low-density lipoprotein cholesterol (LDLC). 

The serum glucose concentration was converted to fasting plasma glucose (FPG) value using the formula from EASD, 2007 [51]: FPG (mmol/L) = −0.137 + 1.047 × serum glucose concentration (mmol/L). Genomic DNA was extracted from whole blood cells via phenol–chloroform technique [52] and stored in deep freezer (minus 70 °C) until further analysis. 

### 4.4. The Measurement of mtDNA-CN 

The measurement of the mtDNA-CN was performed with real-time quantitative PCR (qPCR) using StepOnePlus™ Real-Time PCR System (Applied Biosystems, Thermo Fisher Scientific Inc., USA) based on the method of Ajaz et al., 2015 [53], with modifications. Beta-2-microglobulin (B2M) was used as a single-copy reference gene (nDNA). The quantitative reactions were set separately for mtDNA and B2M in duplicate 96-well plates at identical positions. Several DNA dilutions (1.25, 6.25, 25 and 100 ng) were located at each plate and were used to create a calibration curve and quantify each sample. Standard amplifier software (StepOne™ and StepOnePlus™ Software v2.3.) was used for the calculations. Then, quality control and calculation of the mtDNA/nDNA ratio were performed. A sample would be excluded from further analysis if the amplification curves in three of its replicates had a standard deviation of more than 0.5. Each plate contained a control DNA sample (universal for all plates). To provide comparability between the plates, we tested the relative intensities of the signal from the control. The mtDNA-CN was assessed by the value of the threshold cycle Ct (threshold cycle, the point of intersection of the DNA accumulation schedule and the threshold line), which makes it possible to consider the initial mtDNA-CN and compare samples with each other [54].

### 4.5. Statistical Analysis 

Statistical analysis was conducted using SPSS (v19.0) software package. The dataset includes 371 death cases and 785 controls. 

In the first step, we used descriptive analysis to compare basic characteristics of case and control groups by ANOVA (for continuous variables) and cross tabulations (for categorical variables). In the second step, we applied logistic regression analysis to estimate odds ratios of death per one-decile decrement in mtDNA-CN as continuous variables. The dependent variable was case of all-cause natural death. Model 1 was adjusted for baseline age and sex; Model 2 was adjusted for age, sex, smoking, systolic blood pressure (SBP), total cholesterol (TC), fasting plasma glucose (FPG), triglycerides (TG), BMI and WHR; Model 3 was additionally adjusted for diabetes (instead of FPG), education level and alcohol consumption. In addition, we estimated odds ratios of death by tertiles of mtDNA-CN values using the top tertile as a reference in logistic regression, applying the same covariates for models. The tertile cut points of mtDNA-CN were 1.002 and 1.370. 

Additionally, we conducted several sensitivity analyses. We repeated the analysis stratified by sex using the same Models 1–3. We conducted analyses separately for three categories of causes of death: CVD, cancer and other reasons. Also, to avoid any potential reverse effect of the underlined diseases on the reduction in mtDNA-CN value, we excluded from analysis death cases that occurred within the first 2 years after baseline and 10 years after baseline (median value of follow-up) and repeated logistic regression analyses using the same covariates and models. Finally, we conducted a secondary analysis based on the entire dataset of non-external deaths and controls with available mtDNA-CN measurements without applying any exclusion criteria (*n* = 1474).

## 5. Conclusions

In a nested case–control design, the mtDNA copy number was an inverse predictor of the 15-year risk of all-cause non-external mortality in the middle-aged and elderly population cohort (Caucasoid) in Novosibirsk, West Siberia. 

One half of deaths occurred due to diseases of the circulatory system and the risk of CVD death was increased in the middle tertile of the mtDNA-CN value, pointing toward the inverse relationship between them while without a dose–response effect. A stronger inverse association was found between mtDNA-CN and cancer-related mortality. The easily assessable molecular marker of mtDNA-CN might be a prospective predictor in the prognosis of age-related conditions. However, the growing body of data justifies further research of the deeper mechanisms underlying the association between mtDNA-CN and human diseases.

## Figures and Tables

**Table 1:** Distribution of the baseline covariates among cases of all-cause death and controls (the Russian arm of the HAPIEE study, men and women, baseline survey 2003–2005, 45–69 years).

Covariates	Cases (All-Cause Death)	Controls	*p*-Value
Observed, *n*	371	785	
Age at baseline, years (mean, SD)	59.6 (6.96)	55.7 (6.47)	<0.001
Women, *n* (%)	167 (45.0)	469 (59.7)	0.009
Systolic blood pressure, mmHg (mean, SD)	152.5 (27.58)	137.6 (22.49)	<0.001
Diastolic blood pressure, mmHg (mean, SD)	93.4 (14.56)	88.2 (12.82)	<0.001
Heart rate, beats per min	73.1 (12.17)	70.1 (10.41)	<0.001
Body mass index, kg/m^2^(mean, SD)	28.7 (5.58)	28.0 (4.83)	0.017
Waist/hip ratio, unit (mean, SD)	0.91 (0.08)	0.88 (0.08)	<0.001
Total cholesterol, mmol/L (mean, SD)	6.41 (1.31)	6.31 (1.22)	0.172
LDL cholesterol, mmol/L (mean, SD)	4.16 (1.15)	4.08 (1.09)	0.335
HDL cholesterol, mmol/L (mean, SD)	1.54 (0.38)	1.55 (0.34)	0.565
TG, mmol/L (mean, SD)	1.59 (0.90)	1.47 (0.74)	0.020
Glucose, plasma, mmol/L (mean, SD)	6.27 (2.09)	5.78 (1.08)	<0.001
Hypertension, *n* (%)	273 (73.8)	428 (54.5)	<0.001
HT treatment (among HT), *n* (%)	121 (44.3)	202 (47.2)	0.457
Diabetes mellitus type 2, *n* (%)	59 (15.9)	45 (5.7)	<0.001
DM2 treatment (among DM2), *n* (%)	18 (30.5)	11 (24.4)	0.494
Menopause (women), *n* (%)	151 (90.4)	355 (75.7)	<0.001
Smoking status, *n* (%)			<0.001
Present smoker	140 (37.7)	179 (22.8)	
Former smoker	44 (12.5)	110 (14.0)	
Never smoker	187 (50.4)	496 (63.2)	
Frequency of drinking, *n* (%)			<0.001
5+/week	18 (4.9)	17 (2.2)	
1–4/week	85 (22.9)	181 (23.1)	
1–3/month	86 (23.2)	208 (26.5)	
<1/month	121 (32.6)	308 (39.2)	
Non-drinkers	61 (16.4)	71 (9.0)	
Education, *n* (%)			<0.001
Primary	50 (13.5)	20 (2.5)	
Vocational	82 (22.1)	188 (23.9)	
Middle	156 (42.0)	293 (37.3)	
High	83 (22.4)	284 (36.2)	
Marital status, *n* (%)			0.034
Single	111 (29.9)	189 (24.1)	
Married	260 (70.1)	596 (75.9)	
mtDNA-CN, unit	1.24 (0.60)	1.30 (0.49)	0.098

Presented as mean (SD) or *n* (%); SD—standard deviation; CVD—cardiovascular disease; LDL cholesterol—low-density lipoprotein cholesterol; HDL cholesterol—high-density lipoprotein cholesterol; TG—triglycerides; HT—hypertension; DM2—diabetes mellitus type 2; *p*-value—F-Fisher ANOVA or Pearson Chi-squared test.

**Table 2:** Relationship between all-cause death and mtDNA copy number, per 1-decile decrement in mtDNA copy number (cases, *n* = 371 and controls, *n* = 785; men and women, 15-year follow-up).

Biomarker	*n*, Cases/ Controls	Model 1	Model 2	Model 3
		OR (95%CI)	OR (95%CI)	OR (95%CI)
mtDNA-CN, unit per 1 decile	371/785	1.08 (1.04–1.13)	1.06 (1.01–1.12)	1.06 (1.01–1.11)
*p*-value for trends		<0.001	0.014	0.024
Men *				
mtDNA-CN, unit per 1 decile	204/316	1.07 (1.01–1.15)	1.05 (0.98–1.12)	1.04 (0.97–1.12)
*p*-value for trends		0.023	0.192	0.252
Women *				
mtDNA-CN, unit per 1 decile	167/469	1.09 (1.02–1.17)	1.09 (1.01–1.17)	1.08 (1.01–1.17)
*p*-value for trends		0.010	0.021	0.030

Model 1: age- and sex-adjusted; Model 2: adjusted for age, sex, smoking, SBP, TC, TG, BMI, WHR, GPF; Model 3: adjusted for age, sex, smoking, SBP, TC, BMI WHR, DM2, alcohol, education; * Models 1, 2, 3 stratified by sex (sex excluded from covariates).

**Table 3:** Relationship between all-cause death and mtDNA-CN by tertiles of mtDNA-CN (cases, *n* = 371 and controls, *n* = 785; men and women, 15-year follow-up).

Biomarker	*n*, Cases/Controls	Tertiles	Absolute Difference T3-T2 T2-T1	Model 1	Model 2	Model 3
				OR (95%CI)	OR (95%CI)	OR (95%CI)
All-cause death						
mtDNA-CN, unit	371/785	T3 (ref)		1.0	1.0	1.0
		T2	0.51	2.35 (1.70–3.26)	2.43 (1.71–3.47)	2.50 (1.75–3.59)
		T1	0.45	1.59 (1.16–2.17)	1.44 (1.03–2.02)	1.36 (0.96–1.91)
*p*-value for trends				0.003	0.028	0.064

Model 1: age- and sex-adjusted; Model 2: adjusted for age, sex, smoking, SBP, TC, TG, BMI, WHR, GPF; Model 3: adjusted for age, sex, smoking, SBP, TC, BMI WHR, DM2, alcohol, education.

**Table 4:** Relationship between all-cause death and mtDNA-CN, per 1-decile decrement in mtDNA-CN by causes of death * (cases, *n* = 371 and controls, *n* = 785; men and women, 15-year follow-up).

Biomarker	*n*, Cases/ Controls	Model 1	Model 2	Model 3
		OR (95%CI)	OR (95%CI)	OR (95%CI)
Death from CVD				
mtDNA-CN, unit per 1 decile	189/785	1.02 (0.96–1.09)	0.98 (0.92–1.05)	0.96 (0.90–1.03)
*p*-value for trends		0.448	0.562	0.963
Death from cancer				
mtDNA-CN, unit per 1 decile	138/785	1.20 (1.12–1.29)	1.21 (1.13–1.31)	1.20 (1.11–1.29)
*p*-value for trends		<0.001	<0.001	<0.001
Other death causes				
mtDNA-CN, unit per 1 decile	44/785	1.05 (0.94–1.17)	1.00 (0.89–1.12)	0.99 (0.88–1.13)
*p*-value for trends		0.431	0.945	0.995

Model 1: age- and sex-adjusted; Model 2: adjusted for age, sex, smoking, SBP, TC, TG, BMI, WHR, GPF; Model 3: adjusted for age, sex, smoking, SBP, TC, BMI WHR, DM2, alcohol, education; * Models 1,2,3 stratified by causes of death.

**Table 5:** Relationship between all-cause death and mtDNA-CN by tertiles of mtDNA-CN by causes of death * (cases, *n* = 371 and controls, *n* = 785; men and women, 15-year follow-up).

Biomarker	*n*, Cases/ Controls	Tertiles	Model 1	Model 2	Model 3
			OR (95%CI)	OR (95%CI)	OR (95%CI)
Death from CVD					
mtDNA-CN, unit	189/785	T3 (ref)	1.0	1.0	1.0
		T2	1.96 (1.29–3.00)	1.89 (1.17–3.04)	1.92 (1.18–3.15)
		T1	1.25 (0.83–1.86)	1.01 (0.64–1.59)	0.87 (0.54–1.40)
*p*-value for trends			0.278	0.974	0.594
Death from cancer					
mtDNA-CN, unit	138/785	T3 (ref)	1.0	1.0	1.0
		T2	3.13 (1.96–5.02)	3.75 (2.27–6.21)	3.66 (2.21–6.05)
		T1	2.32 (1.48–3.65)	2.44 (1.52–3.92)	2.29 (1.43–3.68)
*p*-value for trends			<0.001	<0.001	<0.001
Other death causes					
mtDNA-CN, unit	44/785	T3 (ref)	1.0	1.0	1.0
		T2	1.97 (0.90–4.31)	1.45 (0.63–3.31)	1.52 (0.65–3.54)
		T1	1.21 (0.59–2.48)	0.91 (0.43–1.96)	0.91 (0.41–2.03)
*p*-value for trends			0.614	0.798	0.806

Model 1: age- and sex-adjusted; Model 2: adjusted for age, sex, smoking, SBP, TC, TG, BMI, WHR, GPF; Model 3: adjusted for age, sex, smoking, SBP, TC, BMI, WHR, DM2, alcohol, education; * Models 1,2,3 stratified by causes of death.

## Data Availability

The data presented in this study are available in tabulated form on request. The data are not publicly available due to ethical restrictions and project regulations.

## Supplementary Materials

The following supporting information is available online at https://www.mdpi.com/article/10.3390/ijms241310469/s1.
