# Supplementary material for: The Relationship between All-Cause Natural Mortality and Copy Number of Mitochondrial DNA in a 15-Year Follow-Up Study
Source: Int J Mol Sci. 2023 Jun 21;24(13):10469. doi: 10.3390/ijms241310469 (PMC10341474; doi:10.3390/ijms241310469)
Supplement: Supplementary file 1 [file ijms-24-10469-s001.zip › ijms-2457163-supplementary.pdf]

Supplementary material

Supplementary Table S1. Relationship between all-cause death and LTL and mtDNA copy number per 1 decile decrement of each biomarker in a cohort excluding early cases of death within 2 years and within 10 years from baseline (cases and controls, n = 427/799 for LTL, 351/785 for mtDNA-CN; men and women, 15-year follow-up)

Biomarker	n, cases/ controls	Model 1	Model 2	Model 3
		OR (95%CI)	OR (95%CI)	OR (95%CI)
Excluded cases of death within 2 years				
mtDNA-CN, unit per 1 decile	351/785	1.08 (1.03-1.13)	1.06 (1.01-1.12)	1.06 (1.01-1.12)
p-value for trends		0.001	0.016	0.024
Excluded cases of death within 10 years				
mtDNA-CN, unit per 1 decile	216/785	1.07 (1.01-1.13)	1.05 (0.99-1.12)	1.05 (0.99-1.12)
p-value for trends	0.023	0.023	0.094	0.092

Model 1: age- and sex-adjusted; Model 2: adjusted for age, sex, smoking, SBP, TC, TG, BMI, WHR, GPF; Model 3: adjusted for age, sex, smoking, SBP, TC, BMI, WHR, DM2, alcohol, education.
